# Critical Role of microRNA-21 in the Pathogenesis of Liver Diseases

**DOI:** 10.3389/fmed.2020.00007

**Published:** 2020-01-31

**Authors:** Ting Zhang, Zhihong Yang, Praveen Kusumanchi, Sen Han, Suthat Liangpunsakul

**Affiliations:** ^1^Division of Gastroenterology and Hepatology, Department of Medicine, Indiana University School of Medicine, Indianapolis, IN, United States; ^2^Indiana Center for Liver Research, Department of Medicine, Indiana University, Indianapolis, IN, United States; ^3^Roudebush Veterans Administration Medical Center, Indianapolis, IN, United States; ^4^Department of Biochemistry and Molecular Biology, Indiana University School of Medicine, Indianapolis, IN, United States

**Keywords:** miRNA-21, viral hepatitis, non-alcoholic fatty liver disease, alcohol liver disease (ALD), hepatocellular carcinoma

## Abstract

MicroRNAs are small non-coding RNAs that range in length from 18 to 24 nucleotides. As one of the most extensively studied microRNAs, microRNA-21 (miR-21) is highly expressed in many mammalian cell types. It regulates multiple biological functions such as proliferation, differentiation, migration, and apoptosis. In this review, we summarized the mechanism of miR-21 in the pathogenesis of various liver diseases. While it is clear that miR-21 plays an important role in different types of liver diseases, its use as a diagnostic marker for specific liver disease or its therapeutic implication are not ready for prime time due to significant variability and heterogeneity in the expression of miR-21 in different types of liver diseases depending on the studies. Additional studies to further define miR-21 functions and its mechanism in association with each type of chronic liver diseases are needed before we can translate the bedside observations into clinical settings.

## Introduction

MicroRNAs (miRNAs) are small non-coding RNAs with 18–24 nucleotides in length. MiRNAs can bind to target mRNAs and negatively regulate gene expression (1). MiRNAs are transcribed by RNA polymerase II as part of capped and polyadenylated primary transcripts (pri-miRNAs) that can be either protein-coding or non-coding (2). The biogenesis of miRNAs can be regulated either at the transcriptional level by specific transcription factors or at the post-transcriptional level by changes in processing (3, 4). MiRNAs target and regulate essentially all biological processes and cell types, and influence complex programs of gene expression in several cellular processes. Particular miRNAs emerge as principal regulators that control major cell functions in various physiological and pathophysiological settings.

**Figure 1:**
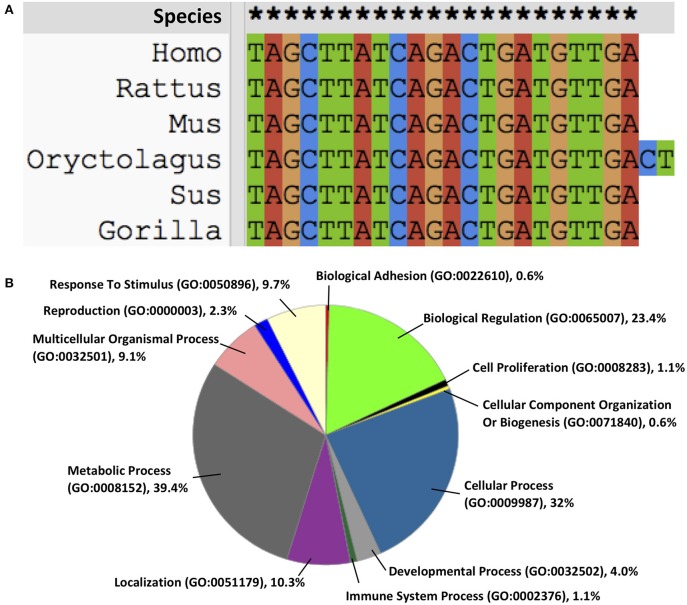
**(A)** miR-21 is highly conserved across the species, **(B)** the functions of miR-21 on intracellular biological processes.

MicroRNA-21 (miR-21) gene is located on chromosome 17 of Homo sapiens and highly conserved (Figure 1A). Its promoter described by Fujita et al. has several conserved enhancer elements including binding sites for activation protein 1 (AP-1; composed of Fos and Jun family proteins), E26 transformation-specific family transcription factor PU1 (Ets/PU1), CCAAT/enhancer binding proteins α (C/EBPα), nuclear factor I (NFI), serum response factor (SRF), p53 and signal transducer and activator of transcription 3 (STAT3) (5, 6). At the cellular level, miR-21 is located in the cytosol (7), extracellular exosome (8), and at the organ level, miR-21 is found in peripheral blood, bone marrow, liver, lung, kidney, Intestine, colon, and thyroid (9). Functionally, miR-21 regulates its targets via interaction with the 3′ untranslated region (UTR) binding involving in post-transcriptional gene silencing (10). It is predicted using computational algorithms that 175 genes involving in biological regulation, cellular and metabolic processes are under regulation of miR-21 [Figure 1B; (11)], however, relatively few have been experimentally validated (Table 1).

**Table 1:** Reported gene targets known to be regulated by miR-21.

**Targets**	**Gene name**	**Mainly function**	**Disease**	**References**
FASLG	Fas ligand	Regulation of the immune system and the progression of cancer	ALD, HCC	(12, 13)
PTEN	Phosphatase and tensin homolog	Regulation of the cell cycle	lung squamous carcinoma, HCC	(14, 15)
TFDP3	Transcription Factor Dp Family Member 3	Regulation of the cell cycle	Lung cancer	(16)
HBP1	HMG-Box Transcription Factor 1	Transcriptional repressor, regulation of the cell cycle	NAFLD and HCC	(17)
HMGCR	3-Hydroxy-3-Methylglutaryl-CoA Reductase	A key enzyme of mevalonate pathway, which produce cholesterol and isoprenoids.	NAFLD	(18)
FABP7	Fatty Acid Binding Protein 7	Fatty acid uptake, transport, and metabolism	NAFLD	(19)
HIF-1a	Hypoxia-inducible factor 1-alpha	A transcriptional regulator of cell response to hypoxia, involving cell survival, tumor invasion, and angiogenesis	ovarian cancer	(20)
PDCD4	Programmed Cell Death 4	Plays a role in apoptosis	breast cancer	(21, 22)
PPARα	Peroxisome proliferator-activated receptor alpha	Regulation of lipid metabolism in liver	NAFLD	(23)
TGF-β	Transforming growth factor, beta	Multifunctional cytokine, regulation of immune cells, cell growth.	spinal cord injury, colon cancer	(24, 25)
SMAD7	SMAD Family Member 7	Inhibitor of the TGF-β signaling	NASH	(26)
IL-12	Interleukin 12	A T cell-stimulating factor, activation of immune response	HCC	(27)
RECK	Reversion-inducing cysteine-rich protein with Kazal motifs precursor	Metalloendopeptidase inhibitor, wnt-protein binding	HCC	(28)
TIMP-3	Tissue inhibitors of metalloproteinases 3	Inhibitor of the matrix metalloproteinases	Liver fibrosis, HCC	(28, 29)

MiR-21 is upregulated in many biological processes, including inflammation, fibrosis, and cancer (5). Increasing evidence has demonstrated the important role of miR-21 in several types of liver diseases. In this current review, we summarized the mechanism of miR-21 in common liver diseases, such as viral hepatitis, non-alcoholic fatty liver disease (NAFLD), alcohol-associated liver disease (ALD), and hepatocellular carcinoma (HCC).

## MiR-21 in Viral Hepatitis

Host miRNAs may target viral genomes or cellular factors, positively or negatively regulating viral infection (30). Viral infections can affect cellular miRNA expression levels and create a favorable environment for their survival and pathogenic effects (30). Serum levels of miR-21 were increased in patients infected with Hepatitis B virus (HBV) (31, 32). Although there was no direct evidence to prove that miR-21 was responsible for HBV infection or replication, some studies showed that miR-21 was essential in the HBV x protein (HBx) induced non-tumor to tumor transformation (27, 31, 33), mechanically through phosphatase and tensin homolog/phosphoinositide 3-kinase/protein kinase B (PTEN/PI3k/Akt) signaling pathway (34).

Hepatitis C virus (HCV) increases the expression of miR-21 in hepatocyte cell lines and primary human hepatocytes (35). Clinical data showed that miR-21 expression in liver tissues was associated with viral load and the level of fibrosis in liver biopsies of patients with HCV infection (36). Chen et al., showed that during HCV infection miR-21 negatively regulated IFN-α signaling by inhibiting myeloid differentiation primary response 88 (MyD88) and Interleukin 1 Receptor Associated Kinase 1 (IRAK1) (37).

## MiR-21 in Non-Alcoholic Fatty Liver Disease (NAFLD)

NAFLD is one of the most common chronic liver diseases which is associated with metabolic syndrome. It represents a broad spectrum of histopathological changes ranging from simple steatosis, steatohepatitis (NASH), and cirrhosis (38, 39). Hepatic miR-21 expression is increased in animal models and patients with NAFLD/NASH (23, 40, 41); however, serum miR-21 levels in NAFLD patients when compared to controls were varied depending on the studies. One study showed that serum miR-21 level was lower in 25 NAFLD patients than those in 12 healthy controls (18), the other study claimed that serum level of miR-21 was higher in patients with NAFLD (42). Several studies showed that miR-21 relies on a complex transcription network to regulate glucose and lipid metabolism in hepatocytes. MiR-21, in part, promotes hepatic lipid accumulation by interacting with several factors, such as sterol regulatory element binding protein (SREBP1) (17, 43), 3-hydroxy-3-methylglutaryl-co-enzyme A reductase (HMGCR) (18), fatty acid binding protein 7 (FABP7) (19). In addition, Calo et al. (44) revealed a new role for miR-21 in hepatocytes in promoting hepatic insulin resistance and steatosis in diet-induced obese mice through regulation of forkhead box protein O1 (Foxo1), insulin induced gene 2 (Insig2), STAT3 and Hepatocyte nuclear factor 4 alpha (HNF4-α). Lack of hepatic miR-21 was sufficient to improve glucose tolerance, insulin sensitivity as well as to prevent hepatic steatosis and fatty acid uptake. MiR-21 also contributes to cell injury, inflammation and fibrosis, through its inhibition of peroxisome proliferator-activated receptor alpha (PPARα) signal pathway (23). Taken together, miR-21 may therefore be implicated at different steps of the NAFLD progression in a cell-specific manner: (1) early steps of lipid accumulation and steatosis onset in hepatocytes and/or (2) inflammation and fibrosis at later stages of the disease (45).

## MiR-21 in Alcohol Associated Liver Disease (ALD)

ALD comprises of histopathological changes similar to those of NAFLD in patients with excessive alcohol use. Several miRNAs are aberrantly expressed after alcohol-induced liver injury. In animal models of mice fed with ethanol via intragastric ethanol feeding (12) or 5 weeks Lieber Decarli ethanol feeding (12), the levels of hepatic miR-21 were found to be differentially overexpressed in mice fed with ethanol compared to pair-fed controls. The induction of hepatic miR-21 is believed to exert its protective effect against liver injury secondary to alcohol. First, overexpression of miR-21 increases cell survival during alcohol-induced liver injury (12). Second, alcoholic hepatitis and alcoholic cirrhosis lead to alterations of tissue repair; a process involving a series of death receptor signaling pathways (46, 47). MiR-21 is a putative mediator of hepatic damage and crucial in tissue repair during alcohol exposure (12). Third, miR-21 may serve as a key regulator of liver regeneration in response to liver injury secondary to alcohol consumption (48). In addition to the findings in animal model, there are 2 lines of evidence supporting the important role of miR-21 in ALD. Integrative miRNA profiling of human liver tissues revealed an important dysregulation of miRNA expression among patients with AH compared to controls (49). Among miRNAs which were differentially expressed from miRNA profiling, hepatic miR-21 was confirmed and validated to be significantly upregulated in patients with AH (49). Despite the evidence suggesting the protective role of miR-21 in ALD, an in-depth analysis to further study the molecular mechanism on the role of miR-21 on the 3 key histological pathologies commonly observed in alcoholic hepatitis; steatosis, inflammation, and fibrosis, are lacking. The processes involving in the spectrum of alcohol-induced liver injury are complex and involved the cross talk between the hepatocytes, kupffer cells (KCs), and stellate cells. Apoptotic hepatocytes secondary to alcohol-induced liver injury promote secretion of inflammatory and pro-fibrogenic cytokines from KCs (47). The role of KCs in the pathogenesis of liver fibrosis has been shown indispensable since macrophage depletion blunts the development of fibrosis (50). As miR-21 is present in the hepatocyte (12) and inflammatory cells/macrophage (51), and stellate cells (52), the specific role of miR-21 from different cell types contributing to ALD pathogenesis should be further studied.

## MiR-21 in Liver Fibrosis and Hepatocellular Carcinoma (HCC)

MiR-21 has been shown to promote fibrogenesis in muscles and various organs including heart, kidneys, lungs, and liver (53). Clinical data also showed that miR-21 expression was up-regulated in liver of patients with biliary atresia-induced liver fibrosis (54). In liver, miR-21 induces fibrosis by activating hepatic stellate cells (HSCs) and collagen synthesis (52, 55, 56). Mechanically, the over expression of miR-21 promotes oxidation, increases in collagen production and activates angiotensin via sprouty RTK Signaling Antagonist 1 (Spry1)/ERK/NF-κB, PTEN/Akt, programmed cell death 4 (PDCD4)/AP-1, Smad7/Smad2/3/NADPH oxidase 4 (NOX4) pathways (52, 57, 58). Recently, research showed that in an methionine choline deficient diet model of NASH-associated liver damage, miR-21 knockout results in decrease of steatosis, inflammation, and lipoapoptosis, with impairment of fibrosis (59). Similarly, in a different study, the loss of miR-21 expression resulted in decreased collagen deposition and expression of fibrotic markers transforming growth factor-β1 and α-smooth muscle actin in bile duct ligation mice model (60). Despite the evidence on the role of miR-21 and fibrosis, a recent study found that antisense inhibition or genetic deletion of miR-21 does not alter HSC activation or liver fibrosis in CCL_4_ induced liver fibrosis mice models (29).

MiR-21 is an “onco-miR,” and miR-21 is frequently up-regulated in human solid malignancies, such as tumors of breast, colon, lung, pancreas, prostate, liver, and stomach (61). MiR-21 is an established survival factor during liver injury and hepatocellular carcinoma development. Clinical data showed that miR-21 was significantly upregulated in both HCC tissues and serum (62–64). Although miR-21 expression in HCC tissues did not predict overall survival (64), studies showed that increased expression of miR-21 was significantly correlated with tumor progression and could be a novel potential biomarker for HCC prognosis (63–65). Mechanically, miR-21 promotes migration and invasion in HCC through the miR-21-PDCD4-AP-1 feedback loop (66). Upregulation of miR-21 can activate phosphatase and tensin homolog (PTEN), which activates phosphatidylinositol 3-kinase signaling to AKT and contributes to progression of HCC (67). Moreover, miR-21 promotes cell migration and invasion of hepatocellular carcinoma by targeting Kruppel Like Factor 5 (KLF5) (68). In addition, HCC cells secreted exosomal miRNA-21 that directly targeted PTEN, leading to activation of pyruvate dehydrogenase kinase 1 (PDK1)/AKT signaling in HSCs; then promoted cancer progression by secreting angiogenic cytokines, including vascular endothelial growth factor (VEGF), matrix metallopeptidase 2 (MMP2), MMP9, basic fibroblast growth factor (bFGF), and transforming growth factor-β (TGF-β) (69). In two separate HCC tumor xenograft models, treatment with specific single-stranded oligonucleotide inhibitors of miR-21 (anti-miRNAs) suppresses HCC growth (70).

## Potential Roles of miR-21 as Diagnostic and Therapeutic Targets for Liver Diseases

While it is clear that miR-21 plays an important role in different types of liver diseases, its use as a diagnostic marker for specific liver disease or its therapeutic implication are not ready for prime time. Circulating miR-21 as a diagnostic marker for disease staging such as in patients with NAFLD yielded contradicting results (18, 42). More importantly, the lack of standard operating procedures and the uniform method to normalize the level of miR-21 with gatekeeping genes are also problematic to adopt to use of miR-21 as the diagnostic tool. Targeting miRNA has previously been conducted for the treatment of hepatitis C infection (36), however, more studies are needed to further explore specific mechanisms of miR-21 in the pathogenesis of various types of liver diseases before its use as a therapeutic intervention.

## Summary

Dysregulation of MiR-21 is common in several types of chronic liver diseases (Table 2). However, in each type of liver disease, there is a variability and heterogeneity in the expression of miR-21 depending on the studies. The underlying explanation may be due to the use of different animal models and lack of standardized procedures and methods to normalize its level. There are several pitfalls in using miR-21 as the therapeutic target or as potential biomarkers for specific types of liver diseases. Additional studies to further define miR-21 functions and its mechanism in association with each type of chronic liver diseases are needed before we can translate the bedside observations into clinical settings.

**Table 2:** Summary of miRNA-21 dysregulation in various liver diseases.

**MiR-21 dysregulation**	**Sample type**	**Detect methods**	**Liver diseases**	**References**
Up-regulation	Human serum	RT-qPCR	HBV	(31, 32)
Up-regulation, correlated with fibrotic stage, viral load	Human liver	RT-qPCR	HCV	(36)
Up-regulation	Cell	RT-qPCR	HCV	(36)
Up-regulation	Mice liver	RT-qPCR	High fat diet model	(23)
Up-regulation	Human liver	microarray	NASH	(23)
Down-regulation	Human serum	RT-qPCR	NAFLD	(18)
Up-regulation	Human serum	RT-qPCR	NAFLD	(42)
Up-regulation	Mice liver	microarray	ALD	(12)
Up-regulation	Human liver	microarray	AH	(49)
Up-regulation	Human liver	RT-qPCR	Liver fibrosis with biliary atresia	(54)
Up-regulation	Human liver, serum	RT-qPCR	HCC	(62, 63)

## Author Contributions

TZ, ZY, and SL contribute article design. TZ, ZY, PK, and SH contribute data collection. TZ contributes first drafts and final submission. SL contributes revision articles.

### Conflict of Interest

The authors declare that the research was conducted in the absence of any commercial or financial relationships that could be construed as a potential conflict of interest.

## Abbreviations

ALD: alcohol-associated liver disease
AP-1: activation protein 1
C/EBPα: CCAAT/enhancer binding proteins α
Ets/PU1: E26 transformation-specific family transcription factor PU1
FABP7: fatty acid binding protein 7
FASLG: Fas ligand
Foxo1: forkhead box O1
HBP1: HMG-Box transcription factor 1
HCC: hepatocellular carcinoma
HMGCR: 3-hydroxy-3-methylglutaryl-coA reductase
HNF4-α: hepatocyte nuclear factor 4 alpha
IL-12: interleukin 12
Insig2: insulin induced gene 2
IRAK1: interleukin 1 Receptor Associated Kinase 1
KC: Kupffer cells
KLF5: Kruppel Like Factor 5
MMP: matrix metallopeptidase
MyD88: myeloid differentiation primary response 88
NAFLD: non-alcoholic fatty liver disease
NASH: non-alcoholic steatohepatitis
NFI: nuclear factor I
NOX4: NADPH oxidase 4
PDCD4: programmed cell death 4
PPARα: peroxisome proliferator-activated receptor alpha
PTEN: phosphatase and tensin homolog
RECK: reversion inducing cysteine rich protein with kazal motifs
SMAD: a family of proteins similar to the gene products of the Drosophila gene 'mothers against decapentaplegic&#8217 (Mad) and the *C. elegans* gene Sma.
Spry1: sprouty RTK signaling antagonist 1
SREBP1: sterol regulatory element binding protein
SRF: serum response factor
STAT3: signal transducer and activator of transcription 3
TFDP3: transcription factor Dp family member 3 TGF-β transforming growth factor-β
TIMP-3: tissue inhibitors of metalloproteinases 3
VEGF: vascular endothelial growth factor.
